# Glial Cell Line-Derived Neurotrophic Factor Family Members Reduce Microglial Activation via Inhibiting p38MAPKs-Mediated Inflammatory Responses

**DOI:** 10.1155/2014/369468

**Published:** 2014-06-09

**Authors:** Uta Rickert, Steffen Grampp, Henrik Wilms, Jessica Spreu, Friederike Knerlich-Lukoschus, Janka Held-Feindt, Ralph Lucius

**Affiliations:** ^1^Department of Anatomy, University of Kiel, Olshausenstraße 40, 24098 Kiel, Germany; ^2^Department of Nephrology and Hypertension, Friedrich-Alexander-University Erlangen-Nuernberg, 91054 Erlangen, Germany; ^3^Department of Neurology, Texas Tech University, 3601 4th Street, Lubbock, TX 79430, USA; ^4^Department of Neurosurgery, University Hospital of Schleswig-Holstein, Campus Kiel, 24105 Kiel, Germany

## Abstract

Previous studies have shown that glial cell line-derived neurotrophic factor (GDNF) family ligands (GFL) are potent survival factors for dopaminergic neurons and motoneurons with therapeutic potential for Parkinson's disease. However, little is known about direct influences of the GFL on microglia function, which are known to express part of the GDNF receptor system. Using RT-PCR and immunohistochemistrym we investigated the expression of the GDNF family receptor alpha 1 (GFR alpha) and the coreceptor transmembrane receptor tyrosine kinase (RET) in rat microglia *in vitro* as well as the effect of GFL on the expression of proinflammatory molecules in LPS activated microglia. We could show that GFL are able to regulate microglia functions and suggest that part of the well known neuroprotective action may be related to the suppression of microglial activation. We further elucidated the functional significance and pathophysiological implications of these findings and demonstrate that microglia are target cells of members of the GFL (GDNF and the structurally related neurotrophic factors neurturin (NRTN), artemin (ARTN), and persephin (PSPN)).

## 1. Introduction

Microglia are distributed throughout the CNS as a network of resting immunocompetent cells derived from the monocyte/macrophage lineage. Alterations in the CNS homeostasis alert microglia and they become rapidly activated in response to injury or the presence of pathogens. Although microglial activation is necessary for host defense and neuroprotection, increased or prolonged activation can have detrimental and neurotoxic effects. By releasing various factors such as cytokines (i.e., interleukins: IL-1*β*, IL-6) or proinflammatory molecules (e.g., prostaglandins, proteolytic enzymes, reactive oxygen intermediates (ROI), or nitric oxide (NO)), [1–3] microglia are able to damage CNS cells. Interestingly, it was demonstrated that microglial inducible nitric oxide synthase (iNOS) as well as IL-1*β* levels is increased in the brain of patients suffering from Parkinson's disease (PD) [4], leading to the hypothesis that the increased levels of iNOS or IL-1*β* may contribute to the pathophysiology of neurodegenerative disorders, especially for PD [5].

In search of new therapeutic agents for neurodegenerative disorders like PD, special interest has been devoted to neurotrophins because of their potential to promote survival and neuritic growth as well as influence the differentiation of several neuronal populations. The neurotrophin glial cell line-derived neurotrophic factor (GDNF) has received lots of attention because it has been shown to be a potent survival factor for dopaminergic midbrain [6, 7] and spinal cord neurons [8]. In some pathological circumstances, such as experimental status epilepticus or experimental traumatic injury, the transcription of GDNF is upregulated in the hippocampus, striatum [9], and spinal cord [10]. Because of the potential protective properties of GDNF in several neurodegenerative disorders such as cerebral ischemia/hypoxia [11] and spinal cord injury [12], GDNF is of special interest for the development of a neurotrophic factor-based therapy for the treatment of neurodegenerative disorders in humans [13].

The GDNF family of ligands (GFL) consists of four structurally related and secreted neurotrophic factors—GDNF, neurturin (NRTN), artemin (ARTN), and persephin (PSPN). GDNF signalling is mediated by a two-component receptor consisting of a GDNF binding domain (*α*) and a signal transducing domain (RET).* In situ* studies revealed that the GDNF receptor is mainly expressed in neurons in the CNS [14, 15]. However, there are also reports that demonstrate the expression of GDNF receptors in microglia: Walker et al. reported the expression of the RET-receptor on microglia in the substantia nigra of both Parkinsonian patients and normal persons by immunohistochemistry [16], while Honda et al. demonstrated the expression in cultivated microglia cells [17].* In vitro* studies have shown that following GDNF binding to GFR*α*1 the resulting complex recruits RET, leading to its activation by dimerization and autophosphorylation at specific cytoplasmic tyrosine residues, thus initiating a number of downstream intracellular pathways [18]. On the other hand, a RET-independent pathway of GDNF signaling that involves the association of GFR*α*1 with the p140^NCAM^ isoform of the neural cell adhesion molecule (NCAM) and subsequent activation of Fyn and FAK kinases has been demonstrated as well as taking place in primary glial cells and neurons [19].

Despite the demonstrated benefits in neuroprotection, there are only few data available describing GDNF-mediated influences on microglia [20, 21]. The neuroprotective effect of GDNF gives rise to the hypothesis that the therapeutic benefit of GDNF in the severed CNS might be at least partly due to an influence on the microglial environment. Although the neurotrophic effect of GDNF on neurons is well established, its action on microglial activities remains to be clarified since this molecule has been considered as a potent agent for CNS treatment.

## 2. Methods

### 2.1. Cultivation of Microglia

For all experiments microglia were prepared from rostral mesencephali and cerebral hemispheres of 2-day-old Wistar rats as described previously [22]. Animals were maintained under constant standard conditions in the “Victor-Hensen” animal house of University of Kiel. Briefly, meninges, hippocampi, and choroid plexus were removed while cortices and mesencephali were minced and enzymatically digested with trypsin (Sigma-Aldrich) and DNAse I (Roche) followed by mechanical dissociation by titration using fire-polished Pasteur pipettes. Suspended cells of two hemispheres or one mesencephalon were plated in a culture flask (75 cm^2^) in 10 mL DMEM (Invitrogen) supplemented with 10% (v/v) FCS (Invitrogen) and 1% (v/v) penicillin/streptomycin (PAA) and cultured in a humidified atmosphere with 5% CO_2_. After 10 days, free floating microglia were daily collected by centrifugation (700 ×g, 5 min) from the medium up to 3-4 weeks. Cell number and viability were estimated by trypan-blue exclusion and only viable cells were used for the experiments. Microglia were seeded out in different concentrations: NO measurement: 200,000 cells/well (96-well plate), real time RT-PCR and ELISA, and 1,000,000 cells/well (12-well plate); Western blot analysis: 1,500,000 cells/well and cells were grown for 24 hrs.

### 2.2. Peptides and Endotoxin LPS

Recombinant human neurotrophic factors GDNF, NRTN, ARTN, and PSPN (all from PeproTech) were freshly prepared by dissolving in distilled water in a concentration of 100 *μ*g/mL and stored as stock solutions at −20°C. For the induction of microglial activation, LPS, from* Salmonella typhimurium* (Sigma), was used.

### 2.3. Cell Stimulation

1,000,000 microglia were preincubated for 30 min with either 50 ng/mL GDNF, 100 g/mL NRTN, 10 ng/mL ARTN, or 5 ng/mL PSPN before LPS (5 ng/mL) was added for further 1, 6, or 24 hrs.

### 2.4. Antibodies and Immunofluorescence Microscopy

The expression of GDNF receptor GFR*α*1 and coreceptor RET was determined by vital immunofluorescence staining. Therefore, the following primary antibodies were used: anti- GFR*α*1 (H-70) sc-10716 (Santa Cruz Biotechnology, 1 : 25) and anti-RET EPR 2871 (Abcam, 1 : 50).

Briefly, 100,000 vital microglia grown on glass cover clips over 24 hrs were washed with PBS, blocked for 60 min at 37°C in 1% (w/v) BSA in DMEM, and then step by step cooled down (30 min room temperature, 10 min 8°C, and 10 min 4°C) in blocking medium. For immune staining, the cells were first washed on ice with washing buffer consisting of 145 mM NaCl, 5.4 mM KCL, 1.8 mM CaCl_2_, 1 mM MgCl_2_, 20 mM glucose, and 20 mM HEPES [23], completed with 1% (w/v) BSA and 1% (v/v) horse serum. The incubation with the primary antibodies was performed in 4% (v/v) horse serum in DMEM (1 hr, 4°C) and the binding of these antibodies was detected by a fluorescent conjugated goat anti-rabbit IgG antibody (Alexa Fluor 488, Invitrogen, 1 : 700 in 4% (v/v) horse serum in DMEM) (1 hr on ice). Afterwards, the cells were washed with washing buffer on ice, fixed in Zamboni's fixative (paraformaldehyde/picric acid) (30 min, room temperature), and nucleoli stained for 3 min with bisbenzimide (Sigma). For fluorescence microscopy cells on each cover slide were mounted using Immo-Mount (Thermo Electron Corporation) and the fluorescence signal was detected with a fluorescence microscope (Zeiss).

### 2.5. Measurement of Nitrite Production

The generation of NO in the supernatants of the cells was determined after 24 hrs by measuring nitrite accumulation in the medium using Griess reagent (1% sulfanilamide and 0.1%* N*-(1-naphthyl)-ethylenediamine dihydrochloride in 5% H_3_PO_4_, Sigma). 100 *μ*L of each culture supernatant and 100 *μ*L Griess reagent were mixed and incubated for 5 min. The absorption was estimated in an automated plate reader (EAR 340 ATTC) at 540 nm. Sodium nitrite (NaNO_2_, Merck) was used to generate a standard curve for quantification. Background nitrite was subtracted from the experimental value. Results were obtained from three separate measurements of identically treated wells/drug, and the data are derived from three or more independent experiments.

### 2.6. Qualitative PCR and Quantitative Real Time PCR (qPCR)

Microglia were washed three times with PBS (4°C). Total RNA was isolated with the phenol chloroform extraction by means of TRIZOL reagent (Invitrogen) according to the manufacturer's guidelines and total RNA was quantified by measuring absorbance at 260 nm. To remove contaminating DNA, RNA was treated with DNase (Promega) and 1 *μ*g of total RNA was reverse- transcribed with RevertAid H Minus M-muLV Reverse Transcriptase (Fermentas) into 20 ng/*μ*L cDNA by random hexamer primer (Amersham Biosciences) and stored at −20°C. Primers of GFR*α*1 used for RT-PCR amplification were 5′-GCACAGCTACGGGATGCTCTTCTG-3′ (sense) and 5′-GTAGTTGGGAGTCATGACTGTGCCAATC-3′ (antisense), primers of coreceptor RET were 5′-ttggtccagtccaacaacaa-3′ (sense) and 5′taggccatgggtaggttcag-3′ (antisense), and primers of GAP-DH as control were 5′-GCACAGTCAAGGCTGAGAATG-3′ (sense) and 5′-TCTTCTGAGTGGCAGTGATGG-3′ (antisense). All primers were manufactured by Eurofins MWG Biotech Operon, Germany.

The PCR solution consisted of 4 *μ*L diluted cDNA, 33,7 *μ*L RNase free water, 2,5 *μ*L of each paired primer, 2 *μ*L dNTP-Mix, 10 × reaction buffer (Eppendorf), and 0,3 *μ*L Hotmaster Taq-Polymerase (Eppendorf). To amplify the DNA the following program (35 cycles) was used: 5 min 94° for activating, 30 sec 94°C for denaturation, 45 sec 60°C for annealing, and 1 min 72°C for extension. 10 *μ*L of the amplified DNA was electrophoresed on a 2% agarose gel and visualized by ethidium bromide, applying 100 bp DNA ladder (Promega).

qPCR was performed in two replicates of each sample using 2 *μ*L of cDNA, 1 *μ*L TaqMan primer probes (assays on demand), 10 *μ*L TaqMan universal PCR primer mastermix (both Applied Biosystems), and 7 *μ*L RNase free water on an ABI Prism 7000 thermocycler. The PCR signal of the target transcript in the treatment groups was related to that of the control by relative quantification. The 2^−ΔΔCT^ method was used to analyze the relative changes in gene expression. The housekeeping gene 18S rRNA was used as internal control to normalize the PCR for the amount of RNA added to the reverse transcription reactions and the target gene expression was normalized to the control. Data are expressed as percent change of gene expression relative to LPS-stimulated cells ( = 100%). TaqMan assays had the following identification numbers: 18s: Hs 99999901; Cox-2 (PtGs2): Rn 00568225; iNOS: Rn 00561646; IL-1*β*: Rn 00580432; IL-6: Rn 00561420; TNF-*α*: Rn 99999017.

### 2.7. Western Blotting

For western blot analysis, microglia were stimulated with GDNF, NRTN, ARTN, and PSP as described, washed twice with ice cold PBS, separately harvested in 100 *μ*L lysis buffer (50 mM Tris (pH 7,5), 100 mM NaCl, 5 mM EDTA, 1% (v/v) triton X-100, 2 mM sodium vanadate, 2,5 mM sodium pyrophosphate, 1 mM *β*-glycerol-phosphate, and 1 mM phenylmethyl-sulfonylfluoride in acetonitrile) by scraping, and homogenized in 1,5 mL tubes. Concentration of isolated proteins (cellular fraction) was determined by Bradford reagent (Bio-Rad Protein Assay, Bio-Rad). 10 *μ*g of isolated proteins was mixed with SDS buffer (2,3% (w/v) SDS; 12,5% (v/v) sample buffer (0,5 M Tris-HCL (pH 6,8) and 0,4% (w/v) SDS in distilled water); 10% (v/v) glycerine and 50 mM DTT) and filled up to 40 *μ*L total volume. Samples were denaturized for 5 min (99°C). Protein aliquots (5 *μ*g each) were resolved by 10% SDS-PAGE and transferred to PDVF membrane (Roth) according to the manufacturer's protocol. The blotted membrane was blocked for 60 min in 5% (w/v) casein dissolved in TBST buffer (20 mM Tris; 0,14 M NaCl; 1 mM EDTA; and 0,1% (w/v) Tween 20). For immunodetection, the membrane was incubated with an antibody against phosphorylated p 38 MAPK (pp 38) (p-p38 (Tyr 182) R sc-7975-R, 200 *μ*g/mL, 1 : 2,000, Santa Cruz Biotechnology) overnight at 4°C. Antibody binding was detected with a HRP-conjugated secondary anti-rabbit antibody (goat anti-rabbit IgG-HRP sc-2004 100 *μ*g/mL, 1 : 30,000, Santa Cruz Biotechnology) and visualized via enhanced chemiluminescence (ECL western blotting detection reagent, Amersham Pharmacia Biotech) according to the manufacturer's protocol. Blot was exposed to chemiluminescence film (HyperfilmTM-ECLTM, Amersham Pharmacia Biotech) and developed. The intensity of each band was analyzed using software PCBAS. As loading control bound pp 38 antibody was stripped and the membrane was incubated with an antibody against p38 MAPK (p38) (p38 MAPK no. D1812, Cell Signaling). Binding was detected with a HRP-conjugated secondary anti-mouse antibody (goat anti-mouse IgG-HRP sc-2031 100 *μ*g/mL, 1 : 20,000, Santa Cruz Biotechnology) and visualized as described above. The results presented are from representative experiments.

### 2.8. Elisa

Cytokine secretion of IL-6 or TNF-*α* to the supernatant was detected after 6 and 24 hrs using a sandwich ELISA (BD) according to the manufacturer's instructions.

In brief, 96-well maxisorp plates (Nunc) were coated with a capture antibody overnight, then blocked with 5% FCS in PBS for 1 h, and washed. Afterwards, a protein standard or samples were added, and plates were incubated for 2 hrs at 37°C. Plates were washed and incubated with a biotinylated detection antibody for 1 h at 37°C. After washing, plates were incubated with HRP-conjugated streptavidin for 30 min at room temperature and washed again. Plates were developed using the tetramethylbenzidine peroxidase substrate system (Thermo Fisher Scientific) and absorbance was measured at 450 nm using an automated plate reader.

### 2.9. Statistical Analysis

All experiments were performed at least three times and the results presented are from representative experiments. The significance of the difference between groups was analyzed using analysis of variance (ANOVA) followed by the Bonferroni test using GraphPad Prism 5 Software. An *α*-level of *P* ≤ 0,05 was used for statistical significance.

## 3. Results

### 3.1. Microglia Express GDNF Receptors GFR*α*1 and RET

**Figure 1:**
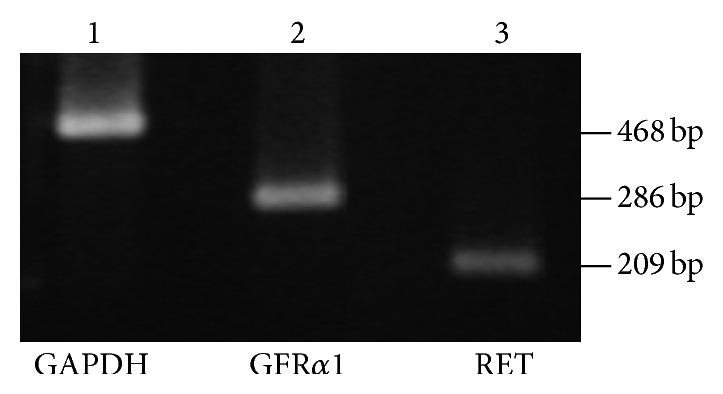
Microglia express GFR-*α*1 and the coreceptor RET. Using qualitative PCR, we investigated whether the GFL receptor GFR-*α*1 and the coreceptor RET are expressed in primary rat microglial cells. Analysis of the PCR products on an ethidium bromide-stained 2% agarose gel demonstrates the expression of both receptors: GFR-*α*1 (lane 2) and RET (lane 3). GAPDH cDNA served as control (lane 1).

**Figure 2:**
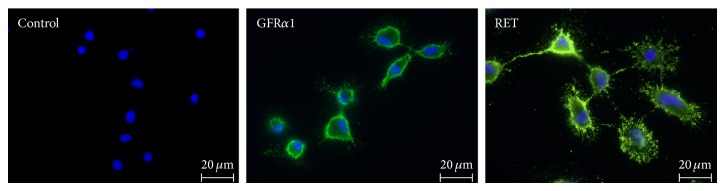
GFR-*α*1 and RET are expressed on microglia. Immunofluorescence staining with specific antibodies shows a diffusely staining pattern of GFR-*α*1 and RET in the cell membrane of primary rat microglial cells. Control cells (omitting of the first antibody) show no specific staining.

To determine whether GDNF receptors are also expressed on primary rat microglia, cells were analyzed by qualitative PCR for mRNA transcripts of GFR*α*1 and RET. As shown in Figure 1, GFR*α*1 and RET mRNA were constitutively expressed in primary rat microglia. In the next step, the occurrence of GDNF receptors was also confirmed by immunofluorescence staining. GFR*α*1 as well as coreceptor RET were immunofluorescence stained in the cell membrane on primary rat microglia (Figure 2).

### 3.2. Inhibition of LPS-Induced Expression of NO and iNOS by GFL. 

To determine whether members of the GFL could inhibit the LPS-induced NO/iNOS synthesis, primary rat microglia cells were pretreated with various GDNF receptor ligands and subsequently stimulated with LPS.

**Figure 3:**
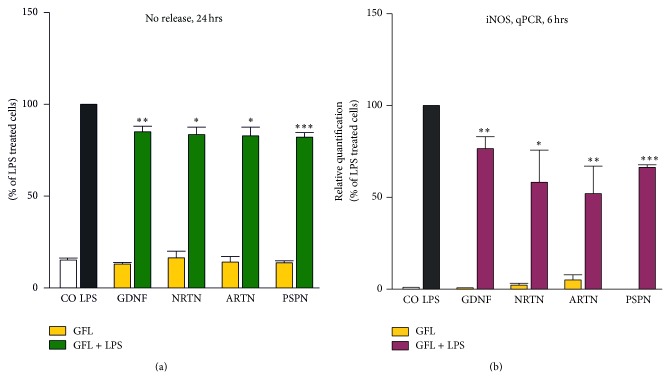
GFL reduce NO synthesis and expression of iNOS in cultivated microglial cells. Microglial cells were pretreated with GFL and afterwards activated with 5 ng/mL LPS. Concentration of nitrite ((a) OD read at 550 nm) in native microglia cell cultures (white bars), microglia stimulated with LPS alone (black bars), with GFL alone (yellow bars), or LPS in combination with GFL members (green) after 24 hrs. The NO reduction is due to downregulation of iNOS (b). Expression of mRNA was analyzed after 6 hrs using TaqMan qPCR compared with the LPS sample. 18sRNA was used as an internal control. Asterisks (^*^ *P* < 0.05, ^**^ *P* < 0.01, and ^***^ *P* < 0.001) indicate a significant difference compared with cells stimulated only with LPS (ANOVA, followed by the Bonferroni test, *n* ≥ 3, LPS = 100%).

After LPS stimulation, NO production by microglia was significantly increased compared to nonstimulated control cells. In the presence of members of the GFL, this effect could be reverted. As shown in Figure 3(a), addition of the GDNF receptor ligands GDNF, NRTN, ARTN, or PSPN significantly reduced the NO production of LPS-stimulated microglia after 24 hrs.

We also tested whether the GFL influence the synthesis of the converting enzyme iNOS. LPS treatment induced the expression of iNOS after 6 hrs in microglia as detected by qPCR. This effect could be significantly reduced by pretreatment of LPS-stimulated primary rat microglia with ligands of the GDNF receptor (Figure 3(b)).

### 3.3. GDNF Receptor Ligands Modulate the Release of Proinflammatory Cytokines

Because excessive release of proinflammatory cytokines is another key feature of activated microglia, we investigated the effect of GFL on LPS-stimulated primary rat microglia.

**Figure 4:**
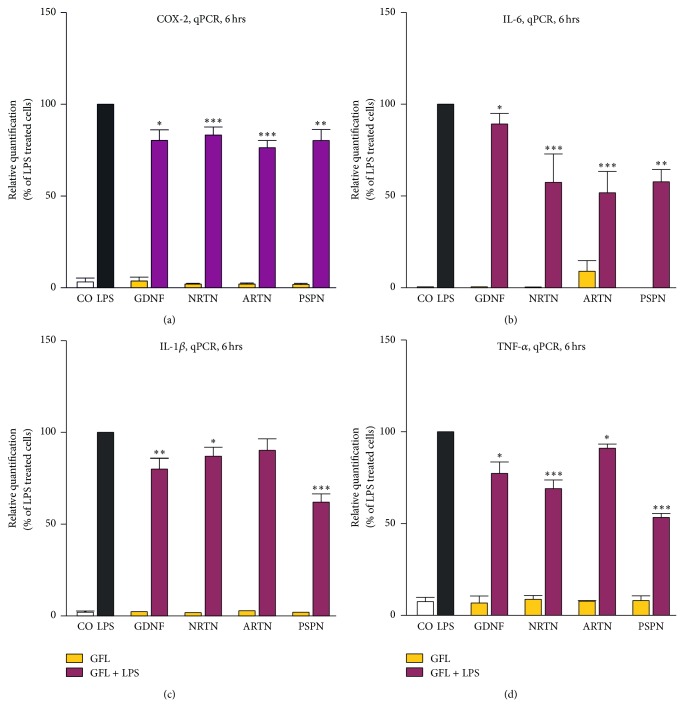
GFL decrease the mRNA expression of proinflammatory cytokines. LPS activated microglial cells were pretreated with GFL. After 6 hrs, mRNA expression was analyzed using TaqMan qPCR of Cox-2 (a), IL-6 (b), IL-1*β* (c), and TNF-*α* (d) and compared with LPS treated microglial cells. 18sRNA was used as an internal control. Asterisks (^*^ *P* < 0.05, ^**^ *P* < 0.01, and ^***^ *P* < 0.001) indicate a significant difference compared with cells stimulated only with LPS (ANOVA, followed by the Bonferroni test, *n* ≥ 3, LPS = 100%).

Pretreatment of primary rat microglia with GFL reduced the LPS-induced expression of proinflammatory cytokines and the inducible rate-limiting enzyme in prostaglandin E(2) synthesis, Cox-2. As shown in Figure 4 the GFL were able to significantly reduce the expression of Cox-2, IL-6, IL-1*β*, and TNF-*α* as compared to LPS-stimulated controls (100%). Interestingly, only the GDNF receptor ligand ARTN did not significantly reduce the level of IL-1*β* transcripts in LPS-stimulated primary rat microglia (Figure 4(c)).

**Figure 5:**
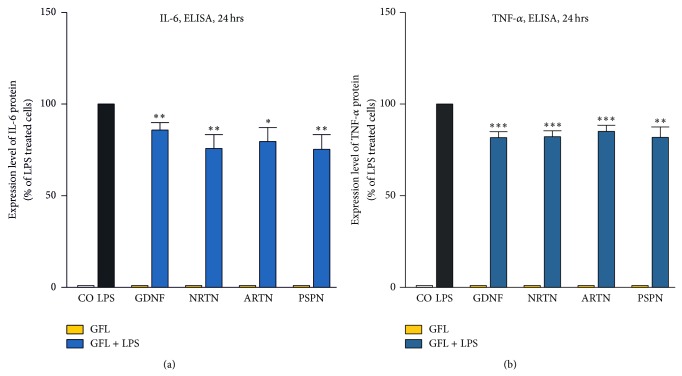
GFL reduced the secretion of IL-6 and TNF-*α*. Supernatants of LPS activated microglial cells pretreated with GFL were analyzed by ELISA for the secretion of IL-6 (a) and TNF-*α* (b) after 24 hrs. The data were assessed from three independent experiments in triplicate. Asterisks (^*^ *P* < 0.05, ^**^ *P* < 0.01, and ^***^ *P* < 0,001) indicate a significant difference compared with cells stimulated with LPS (ANOVA, followed by the Bonferroni test, *n* ≥ 3, LPS = 100%).

Furthermore, we also examined whether the GFL also influence the secretion of the proinflammatory cytokines IL-6 and TNF-*α* into the cell culture supernatants. Surprisingly, GDNF and ARTN have no influence on the secretion of IL-6 after 6 hrs, and NTRN and ARTN have no influence on the TNF-*α* secretion after 6 hrs (data not shown) although they significantly reduced the level of IL-6 or TNF-*α* mRNA transcripts (compare Figure 4). In contrast, all tested GFL reduced protein concentrations of IL-6 (Figure 5(a)) and TNF-*α* (Figure 5(b)) in the supernatants of LPS activated microglia after 24 hrs.

### 3.4. GFL Suppress the Phosphorylation of p38

**Figure 6:**
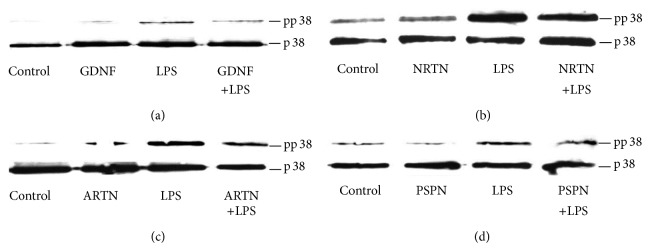
GFL trigger p38-MAPK pathways in rat microglial cells. We investigated changes in the phosphorylation state of mitogen-activated protein kinase (MAPK) p38 in rat microglia stimulated for 1 hr. GDNF (a), NRTN (b), ARTN (c), and PSPN (d) increase phosphorylation of p38, whereas addition of GFL decreases the signaling mechanism. Figure 6 shows representative images of the western blot analysis from 3 independent experiments.

To determine whether the GFL mediated inhibition of microglial activation is due to alterations in the regulation of the p38 MAPK pathway, the effects of GFL ligands on the phosphorylation of p38 finally were examined. All GFL inhibited the phosphorylation of p38 in LPS-stimulated primary rat microglia whereby pp38 in LPS/GFL costimulated cells was downregulated (Figure 6).

## 4. Discussion

There has been a considerable interest in neuroprotective therapies using trophic factors to alleviate the symptoms of PD. Despite the demonstrated benefits in neuroprotection, there is no data available concerning GDNF-mediated influences on the glial—especially microglial environment in the CNS. This could be of interest because neuroprotective effects could be mediated via interference with proinflammatory microglial functions. Since GDNF and NTNR have been considered as potent agents for CNS treatment [24], we addressed whether members of the GFL affect proinflammatory microglial functions.

In the present study we confirmed that rat microglia* in vitro* express GFR*α*1 and transmembrane c-RET tyrosine kinase (RET) [17]. Honda et al. suggested that GDNF is able to regulate certain functions of microglia. However, influences on neuroinflammatory functions were not investigated by them. Next, we used various biochemical analyses to examine whether ligands at the GDNF receptors are able to influence microglial activities, including the production of proinflammatory mediators. We found that the GFL members GDNF, NRTN, ARTN, and PSPN are able to reduce the production of microglial nitric oxide and mRNA levels of IL-1*β*, TNF-*α*, IL-6, and Cox-2. Therefore, the present study provides unequivocal evidence that members of the GFL interfere with the synthesis and release of proinflammatory and neurotoxic molecules generated by activated microglia* in vitro*. This could lead to a reduction of the proinflammatory response of activated microglia with subsequent neuroprotection. For example, NO is neurotoxic due to inhibition of complexes 1 and 2 of the respiratory chain, and iNOS inhibitors are able to prevent mitochondrial injury [25]. Moreover, it reacts with superoxide anion to generate peroxynitrite, a highly reactive molecule capable of oxidizing proteins, lipids, and DNA, which causes striatal neurodegeneration in a mouse model* in vivo* [26]. In macrophages, NO is synthesized in the presence of LPS in large quantities from L-arginine in a calcium independent way by iNOS, which is controlled at least in part via NF-*κ*B driven gene transcription. As mentioned earlier, it has been suggested that a microglial derived NO overload may be one of the crucial elements which promote neuronal damage in acute and chronic CNS degenerative diseases [27]. In addition to the inhibition of NO production, we also determined the release of IL-6 and TNF-*α*. The cytokine TNF-*α* is an important factor in the regulation of apoptotic cell death. TNF-*α* immunoreactive glial cells have been detected in the substantia nigra (SN) and immunoreactivity for TNF receptors was found in cell bodies and processes of most dopaminergic neurons of Parkinsonian patients [28]. It is known that dopaminergic neurons are more vulnerable to TNF-*α* than other neurons [29]. Moreover, the cytokines TNF-*α* and IL-6 are essential players in cerebral inflammation and neurodegeneration (reviewed by Owens et al. [30]).

Prostaglandins may also play a significant role in PD as a cytotoxic mediator of inflammation. For example, Cox-2 activity is upregulated in LPS-stimulated microglia* in vitro* [31], and the pharmacological inhibition of Cox-2 activity protects nigral dopaminergic neuronal loss and decreases microglial activation* in vivo *[32].

While microglia may contribute to the pathogenesis of neurodegeneration, such as in multiple sclerosis [33], Alzheimer's disease [34], and acquired immunodeficiency syndrome-associated dementia [35], their role in PD [36] is most interesting in the context of these results, since GDNF has been used in clinical trials to protect degenerating dopamine neurons as well as promote regeneration of the nigrostriatal dopamine system [37, 38]. In animal models of PD, direct bolus administration of either GDNF or NRTN prevents dopaminergic degeneration (for review see [39]). A component of the GDNF receptor complex, the protooncogene RET, is expressed in substantia nigra neurons of neurologically normal autopsied patients, with persisting expression in surviving neurons in PD. It is noteworthy that this expression was also found on microglia in the PD cases [16]. Activated microglia are a common feature in areas of the SN affected by PD pathology [40]. Moreover, the substantia nigra has an extremely high density of microglia [41], so it is possible that the beneficial effects of GDNF may be partially mediated through an anti-inflammatory effect on microglia. Our results are partly confirmed by a recent work from Rocha et al. [42]. They identified astrocytes as a possible endogenous source of GDNF and suggested that astrocyte derived GDNF can protect from neurodegeneration through inhibition of neuroinflammation. On the other hand, activation of microglia seems to block the secretion of GDNF by microglia itself [43], leading to a loss of anti-inflammatory capacity.

## 5. Conclusion

In conclusion, this study points out the role of GFL in controlling microglial activation. Moreover, the p38-MAPK signaling pathway may be involved in the GDNF-induced mechanism for the regulation of microglial activities. The importance of microglial p38 MAPK was recently shown by Xing et al. [44]. The authors could clearly demonstrate that LPS-induced activation of microglial p38*α* MAPK signaling leads to neuron death which is mediated through upregulation of the proinflammatory cytokine TNF-*α*.

This study provides evidence that exogenous GDNF administration may not only have a protective effect on neurons, but may also have a modulatory role in microglial activities.

## Abbreviations

ARTN: Artemin
Co: Control
Cox-2: Cyclooxygenase-2
GDNF: Glial cell line-derived neurotrophic factor
GFL: Glial cell line-derived neurotrophic factor family ligands
GFR*α*1: GDNF family receptor alpha 1
IL-1*β*: Interleukin-1 beta
IL-6: Interleukin-6
iNOS: Inducible NO synthase
LPS: Lipopolysaccharide
NCAM: Neural cell adhesion molecule
NO: Nitric oxide
NRTN: Neurturin
PD: Parkinson's disease
PSPN: Persephin
qPCR: Quantitative PCR
RET: Receptor tyrosine kinase
ROIs: Reactive oxygen intermediates
SN: Substantia nigra
TGF-*β*: Transforming growth factor-*β*
TNF-*α*: Tumour necrosis factor-alpha.
